# Ticks (Ixodida: Argasidae, Ixodidae, Khimairidae, and Nuttalliellidae) of the world

**DOI:** 10.1186/s13071-026-07493-z

**Published:** 2026-06-14

**Authors:** Filipe Dantas-Torres, Ben J. Mans

**Affiliations:** 1 Department of Immunology, Aggeu Magalhães Institute, Oswaldo Cruz Foundation (Fiocruz), Recife, Pernambuco 50740-465 Brazil; 2 Epidemiology, Parasites and Vectors, Agricultural Research Council-Onderstepoort Veterinary Research, Onderstepoort, 0110 South Africa; 3 Department of Zoology and Entomology, University of the Free State, Bloemfontein, 9300 South Africa; 4 Department of Life and Consumer Sciences, University of South Africa, Florida, 1710 South Africa

**Keywords:** Ticks, Families, Genera, Species, Classification, Taxonomy

## Abstract

The taxonomy of ticks (order Ixodida) has changed significantly over the past few decades. Many species have been described, synonymized, invalidated, or reinstated. The genus-level classification of ticks in the families Argasidae, Ixodidae, and Nuttalliellidae has also been revised. Here, we list 1033 tick species recognized as valid, including 21 fossil species. These ticks are grouped into four families: Argasidae (17 genera, 221 species), Ixodidae (20 genera, 800 species), Khimairidae (one genus, one species), and Nuttalliellidae (three genera, 11 species). An updated classification of tick genera is also provided.

**Graphical Abstract:**

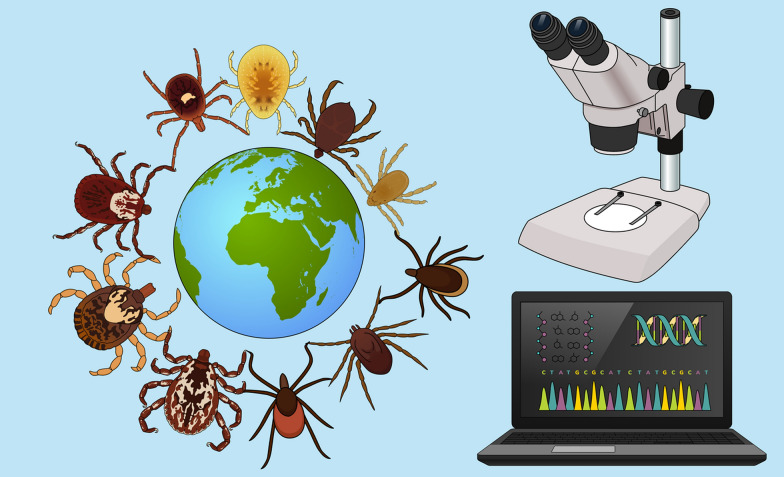

## Background

Ticks (order Ixodida) have been known since ancient times [1, 2]. For millions of years, ticks have roamed the planet, evolving as parasites of vertebrate hosts and feeding on blood for survival [3]. Through blood feeding, ticks can transmit a wide range of pathogens that may affect animals, humans, or both [4]. New tick-borne pathogens continue to be discovered, thanks to advancements in molecular biology [5–7]. Refined morphological and advanced molecular research have also revolutionized tick taxonomy and systematics. In fact, many tick species have been described recently, while others have been synonymized, invalidated, or reinstated. Similarly, the genus-level classification of ticks has changed significantly due to advances in mitochondrial genomes and phylogenetic analyses. As a result, previous lists of tick species of the world are now outdated.

Here, we present a list of 1033 tick species recognized as valid. An updated classification of tick genera is also included.

## Brief historical account of previous lists of valid tick names

One of the earliest comprehensive lists was presented by Keirans [8], who listed 850 valid species or subspecies, grouped into three families and 19 genera. Camicas et al. [9] published a comprehensive book on ticks of the world, listing 869 valid species or subspecies. This book remained, for many years, one of the most informative sources of tick names, including their synonyms. Subsequent comprehensive lists of world tick species have been published [10–14], while others [15, 16] have focused on particular tick families or subjects, such as controversial names [17], species descriptions and redescriptions [18], geographical distribution [19], or type repositories [20]. These lists are content-rich, essential resources for tick taxonomists and anyone working with ticks.

The last comprehensive lists of valid tick species were published in 2010 [13] and 2018 [14]. Since then, some workers have compiled lists of valid argasid [16], or ixodid [19, 20] species, but a comprehensive, up-to-date list remains unavailable.

The last list of valid argasid species was published in 2019 [16]. Since then, additional argasid species have been described, while others have been placed in synonymy or classified as *nomina nuda*. Moreover, the genus-level classification, already updated in Mans et al. [16], has continued to change [21–23]. Similarly, the most recent lists of the family Nuttalliellidae included only one genus and species [13, 14], but the family now includes multiple genera and species [24, 25]. Other tick families were also created, including Deinocrotonidae, which was included in the last list of ticks of the world [14]. However, it is now considered invalid [25]. Several genus-level changes were also observed in the family Ixodidae [26–28]. This highlights the need for an updated list of tick species worldwide, including valid species and genera.

## Contemporary tick classification and a valid species list

Ticks belong to the phylum Arthropoda, subphylum Chelicerata, class Arachnida, order Ixodida, and superfamily Ixodoidea. Currently, four families are recognized as valid: Argasidae, Ixodidae, Khimairidae, and Nuttalliellidae. The family Deinocrotonidae was described in 2017 to include the new genus *Deinocroton* [29], but it was recently synonymized with Nuttalliellidae [25]. The families and genera (with their species counts) are listed in Table 1.

**Table 1:** Tick families and genera (listed alphabetically)

Argasidae	Ixodidae	Khimairidae	Nuttalliellidae
*Alectorobius* (64)	*Africaniella* (2)	*Khimaira* (1)	*Deinocroton* (4)
*Alveonasus* (8)	*Alloceraea* (7)		*Legionaris* (1)
*Antricola* (16)	*Amblyomma* (139)		*Nuttalliella* (6)
*Apanaskevichiella* (1)	*Anomalohimalaya* (3)		
*Argas* (44)	*Archaecroton* (2)		
*Australpavlovskyella* (1)	*Bothriocroton* (8)		
*Carios* (8)	*Compluriscutula* (1)		
*Chiropterargas* (4)	*Cornupalpatum* (1)		
*Navis* (1)	*Cosmiomma* (1)		
*Nothoaspis* (3)	*Cryptocroton* (1)		
*Ogadenus* (1)	*Dermacentor* (45)		
*Ornithodoros* (47)	*Haemaphysalis* (173)		
*Otobius* (2)	*Hyalomma* (27)		
*Proknekalia* (3)	*Ixodes* (291)		
*Reticulinasus* (12)	*Margaropus* (3)		
*Secretargas* (3)	*Nosomma* (2)		
*Subparmatus* (3)	*Rhipicentor* (2)		
	*Rhipicephalus* (90)		
	*Robertsicus* (1)		
	*Sharifiella* (1)		

Species numbers are shown in parentheses

The present list is based on the most recent lists [16, 19, 20], as of April 2026, with the addition of recent species descriptions and reinstatements, as indicated in the sections below. We also updated the classification of tick families and genera based on the latest developments in tick taxonomy and systematics [16, 21, 22, 25, 26, 28, 30]. We do not aim to cover all controversial species, but we provide brief comments on specific ones that have recently been the subject of taxonomic discussion.

## The family Argasidae

The family Argasidae comprises 17 genera and 221 species. Table 2 lists the valid species of Argasidae. These species include 216 species listed in Mans et al. [16], excluding two species considered *nomina nuda* (*Alectorobius jul* (Schulze, 1940) and *Ornithodoros nattereri* Warburton, 1927) [31], a species placed in synonymy (*Antricola inexpectata* Estrada-Peña, Barros-Battesti and Venzal, 2004, synonymized with *Antricola guglielmonei* Estrada-Peña, Barros-Battesti and Venzal, 2004) [32], and adding the following eight described species: *Alectorobius cerradoensis* (Muñoz-Leal, Martins and Labruna, 2020), *Alectorobius montensis* (Venzal, Mangold and Nava, 2019), *Alectorobius octodontus* (Muñoz-Leal, González-Acuña and Venzal, 2020), *Alectorobius tabajara* (Muñoz-Leal and Labruna, 2021), *Ornithodoros huajianensis* Sun, Xu, Liu and Wu, 2019, *Ornithodoros daga* Muñoz-Leal, 2024, *Ornithodoros improvisus* Muñoz-Leal and Venzal, 2023, and *Ornithodoros pakistanensis* Ali, Chitimia-Dobler, Muñoz-Leal and Mans, 2024 [33–40].

**Table 2:** List of valid genera and species of soft ticks (family Argasidae)

No	Species
1	*Alectorobius amblus* (Chamberlin, 1920)
2	*Alectorobius antiquus* (Poinar, 1995)*
3	*Alectorobius aragaoi* (Fonseca, 1960)
4	*Alectorobius atacamensis* (Muñoz-Leal, Venzal and González-Acuña, 2016)
5	*Alectorobius azteci* (Matheson, 1935)
6	*Alectorobius brodyi* (Matheson, 1935)
7	*Alectorobius capensis* (Neumann, 1901)
8	*Alectorobius casebeeri* (Jones and Clifford, 1972)
9	*Alectorobius cavernicolous* (Dantas-Torres, Venzal and Labruna, 2012)
10	*Alectorobius cerradoensis* (Muñoz-Leal, Martins and Labruna, 2020)
11	*Alectorobius cheikhi* (Vermeil, Marjolet and Vermeil, 1997)
12	*Alectorobius chironectes* (Jones and Clifford, 1972)
13	*Alectorobius clarki* (Jones and Clifford, 1972)
14	*Alectorobius collocaliae* (Hoogstraal, Kadarsan, Kaiser and Van Peenen, 1974)
15	*Alectorobius concanensis* (Cooley and Kohls, 1941)
16	*Alectorobius coniceps* (Canestrini, 1890)
17	*Alectorobius cyclurae* (de la Cruz, 1984)
18	*Alectorobius darwini* (Kohls, Clifford and Hoogstraal, 1969)
19	*Alectorobius denmarki* (Kohls, Sonenshine and Clifford, 1965)
20	*Alectorobius dugesi* (Mazzotti, 1943)
21	*Alectorobius dusbabeki* (Černý, 1967)
22	*Alectorobius dyeri* (Cooley and Kohls, 1940)
23	*Alectorobius echimys* (Kohls, Clifford and Jones, 1969)
24	*Alectorobius elongatus* (Kohls, Sonenshine and Clifford, 1965)
25	*Alectorobius eptesicus* (Kohls, Clifford and Jones, 1969)
26	*Alectorobius faccinii* (Barros-Battesti, Landulfo and Luz, 2015)
27	*Alectorobius fonsecai* (Labruna and Venzal, 2009)
28	*Alectorobius galapagensis* (Kohls, Clifford and Hoogstraal, 1969)
29	*Alectorobius guaporensis* (Nava, Venzal and Labruna, 2013)
30	*Alectorobius hasei* (Schulze, 1935)
31	*Alectorobius jerseyi* (Klompen and Grimaldi, 2001)*
32	*Alectorobius kelleyi* (Cooley and Kohls, 1941)
33	*Alectorobius knoxjonesi* (Jones and Clifford, 1972)
34	*Alectorobius kohlsi* (Guglielmone and Keirans, 2002)
35	*Alectorobius lahillei* (Venzal, González-Acuña and Nava, 2015)
36	*Alectorobius maritimus* (Vermeil and Marguet, 1967)
37	*Alectorobius marmosae* (Jones and Clifford, 1972)
38	*Alectorobius microlophi* (Venzal, Nava and González-Acuña, 2013)
39	*Alectorobius mimon* (Kohls, Clifford and Jones, 1969)
40	*Alectorobius montensis* (Venzal, Mangold and Nava, 2019)
41	*Alectorobius muesebecki* (Hoogstraal, 1969)
42	*Alectorobius natalinus* (Černý and Dusbábek, 1967)
43	*Alectorobius octodontus* (Muñoz-Leal, González-Acuña and Venzal, 2020)
44	*Alectorobius peropteryx* (Kohls, Clifford and Jones, 1969)
45	*Alectorobius peruvianus* (Kohls, Clifford and Jones, 1969)
46	*Alectorobius puertoricensis* (Fox, 1947)
47	*Alectorobius quilinensis* (Venzal, Nava and Mangold, 2012)
48	*Alectorobius rietcorreai* (Labruna, Nava and Venzal, 2016)
49	*Alectorobius rioplatensis* (Venzal, Estrada-Peña and Mangold, 2008)
50	*Alectorobius rondoniensis* (Labruna, Terassini, Camargo, Brandão, Ribeiro and Estrada-Peña, 2008)
51	*Alectorobius rossi* (Kohls, Sonenshine and Clifford, 1965)
52	*Alectorobius rudis* (Karsch, 1880)
53	*Alectorobius saraivai* (Muñoz-Leal and Labruna, 2017)
54	*Alectorobius sawaii* (Kitaoka and Suzuki, 1973)
55	*Alectorobius spheniscus* (Hoogstraal, Wassef, Hays and Keirans, 1985)
56	*Alectorobius stageri* (Cooley and Kohls, 1941)
57	*Alectorobius tabajara* (Muñoz-Leal and Labruna, 2021)
58	*Alectorobius tadaridae* (Černý and Dusbábek, 1967)
59	*Alectorobius talaje* (Guérin-Méneville, 1849)
60	*Alectorobius tiptoni* (Jones and Clifford, 1972)
61	*Alectorobius tuttlei* (Jones and Clifford, 1972)
62	*Alectorobius xerophylus* (Venzal, Mangold and Nava, 2015)
63	*Alectorobius yumatensis* (Cooley and Kohls, 1941)
64	*Alectorobius yunkeri* (Keirans, Clifford and Hoogstraal, 1984)
65	*Alveonasus acinus* (Whittick, 1938)
66	*Alveonasus buettikeri* (Vial and Camicas, 2009)
67	*Alveonasus canestrinii* (Birula, 1895)
68	*Alveonasus cooleyi* (McIvor, 1941)
69	*Alveonasus delanoei* (Roubaud and Colas-Belcour, 1931)
70	*Alveonasus eboris* (Theiler, 1959)
71	*Alveonasus foleyi* (Parrot, 1928)
72	*Alveonasus lahorensis* (Neumann, 1908)
73	*Antricola armasi* de la Cruz and Estrada-Peña, 1995
74	*Antricola centralis* de la Cruz and Estrada-Peña, 1995
75	*Antricola cernyi* de la Cruz, 1978
76	*Antricola coprophilus* (McIntosh, 1935)
77	*Antricola delacruzi* Estrada-Peña, Barros-Battesti and Venzal, 2004
78	*Antricola granasi* de la Cruz, 1973
79	*Antricola guglielmonei* Estrada-Peña, Barros-Battesti and Venzal, 2004
80	*Antricola habanensis* de la Cruz, 1976
81	*Antricola hummelincki* de la Cruz and Estrada-Peña, 1995
82	*Antricola marginatus* (Banks, 1910)
83	*Antricola martelorum* de la Cruz, 1978
84	*Antricola mexicanus* Hoffmann, 1958
85	*Antricola naomiae* de la Cruz, 1978
86	*Antricola occidentalis* de la Cruz, 1978
87	*Antricola siboneyi* de la Cruz and Estrada-Peña, 1995
88	*Antricola silvai* Černý, 1967
89	*Apanaskevichiella macmillani* (Hoogstraal and Kohls, 1966)
90	*Argas abdussalami* Hoogstraal and McCarthy, 1965
91	*Argas africolumbae* Hoogstraal, Kaiser, Walker, Ledger, Converse and Rice, 1975
92	*Argas arboreus* Kaiser, Hoogstraal and Kohls, 1964
93	*Argas assimilis* Teng and Song, 1983
94	*Argas beijingensis* Teng, 1983
95	*Argas beklemischevi* Pospelova-Shtrom, Vasil´yeva and Semashko, 1963
96	*Argas brevipes* Banks, 1908
97	*Argas bureschi* Dryenski, 1957
98	*Argas cooleyi* Kohls and Hoogstraal, 1960
99	*Argas cucumerinus* Neumann, 1901
100	*Argas dalei* Clifford, Keirans, Hoogstraal and Corwin, 1976
101	*Argas delicatus* Neumann, 1910
102	*Argas dulus* Keirans, Clifford and Capriles, 1971
103	*Argas falco* Kaiser and Hoogstraal, 1974
104	*Argas giganteus* Kohls and Clifford, 1968
105	*Argas gilcolladoi* Estrada-Peña, Lucientes and Sánchez, 1987
106	*Argas hermanni* Audouin, 1826
107	*Argas himalayensis* Hoogstraal and Kaiser, 1973
108	*Argas japonicus* Yamaguti, Clifford and Tipton, 1968
109	*Argas keiransi* Estrada-Peña, Venzal and González-Acuña, 2003
110	*Argas lagenoplastis* Froggatt, 1906
111	*Argas latus* Filippova, 1961
112	*Argas lowryae* Kaiser and Hoogstraal, 1975
113	*Argas macrostigmatus* Filippova, 1961
114	*Argas magnus* Neumann, 1896
115	*Argas miniatus* Koch, 1844
116	*Argas monachus* Keirans, Radovsky and Clifford, 1973
117	*Argas monolakensis* Schwan, Corwin and Brown, 1992
118	*Argas moreli* Keirans, Hoogstraal and Clifford, 1979
119	*Argas neghmei* Kohls and Hoogstraal, 1961
120	*Argas nullarborensis* Hoogstraal and Kaiser, 1973
121	*Argas persicus* (Oken, 1818)
122	*Argas polonicus* Siuda, Hoogstraal, Clifford and Wassef, 1979
123	*Argas radiatus* Railliet, 1893
124	*Argas reflexus* (Fabricius, 1794)
125	*Argas ricei* Hoogstraal, Kaiser, Clifford and Keirans, 1975
126	*Argas robertsi* Hoogstraal, Kaiser and Kohls, 1968
127	*Argas sanchezi* Dugès, 1887
128	*Argas streptopelia* Kaiser, Hoogstraal and Horner, 1970
129	*Argas theilerae* Hoogstraal and Kaiser, 1970
130	*Argas tridentatus* Filippova, 1961
131	*Argas vulgaris* Filippova, 1961
132	*Argas walkerae* Kaiser and Hoogstraal, 1969
133	*Argas zumpti* Hoogstraal, Kaiser and Kohls, 1968
134	*Australpavlovskyella gurneyi* (Warburton, 1926)
135	*Carios australiensis* (Kohls and Hoogstraal, 1962)
136	*Carios daviesi* (Kaiser and Hoogstraal, 1973)
137	*Carios dewae* (Kaiser and Hoogstraal, 1974)
138	*Carios macrodermae* (Hoogstraal, Moorhouse, Wolf and Wassef, 1977)
139	*Carios pusillus* (Kohls, 1950)
140	*Carios quadridentatus* Heath, 2012
141	*Carios sinensis* (Jeu and Zhu, 1982)
142	*Carios vespertilionis* Latreille, 1796
143	*Chiropterargas boueti* (Roubaud and Colas-Belcour, 1933)
144	*Chiropterargas ceylonensis* (Hoogstraal and Kaiser, 1968)
145	*Chiropterargas confusus* (Hoogstraal, 1955)
146	*Chiropterargas cordiformis* (Hoogstraal and Kohls, 1967)
147	*Navis striatus* (Bedford, 1932)
148	*Nothoaspis amazoniensis* Nava, Venzal and Labruna, 2010
149	*Nothoaspis reddelli* Keirans and Clifford, 1975
150	*Nothoaspis setosus* Kohls, Clifford and Jones, 1969
151	*Ogadenus brumpti* (Neumann, 1907)
152	*Ornithodoros alactagalis* Issaakjan, 1936
153	*Ornithodoros apertus* Walton, 1962
154	*Ornithodoros arenicolous* Hoogstraal, 1953
155	*Ornithodoros asperus* Warburton, 1918
156	*Ornithodoros brasiliensis* Aragão, 1923
157	*Ornithodoros cholodkovskyi* Pavlovsky, 1930
158	*Ornithodoros compactus* Walton, 1962
159	*Ornithodoros coriaceus* Koch, 1844
160	*Ornithodoros costalis* Diatta, Bouattour, Durand, Renaud and Trape, 2013
161	*Ornithodoros daga* Muñoz-Leal, 2024
162	*Ornithodoros eremicus* Cooley and Kohls, 1941
163	*Ornithodoros erraticus* (Lucas, 1849)
164	*Ornithodoros furcosus* Neumann, 1908
165	*Ornithodoros graingeri* Heisch and Guggisberg, 1953
166	*Ornithodoros grenieri* Klein, 1965
167	*Ornithodoros hermsi* Wheeler, Herms and Meyer, 1935
168	*Ornithodoros huajianensis* Sun, Xu, Liu and Wu, 2019
169	*Ornithodoros improvisus* Muñoz-Leal and Venzal, 2023
170	*Ornithodoros indica* Rau and Rao, 1971
171	*Ornithodoros kairouanensis* Trape, Diatta, Bouattour, Durand and Renaud, 2013
172	*Ornithodoros kalahariensis* Bakkes, de Klerk and Mans, 2018
173	*Ornithodoros marocanus* Velu, 1919
174	*Ornithodoros merionesi* Trape, Diatta, Belghyti, Sarih, Durand and Renaud, 2013
175	*Ornithodoros moubata* (Murray, 1877)
176	*Ornithodoros nicollei* Mooser, 1932
177	*Ornithodoros noorsveldensis* Bakkes, de Klerk and Mans, 2018
178	*Ornithodoros normandi* Larrousse, 1923
179	*Ornithodoros occidentalis* Trape, Diatta, Durand and Renaud, 2013
180	*Ornithodoros pakistanensis* Ali, Chitimia-Dobler, Muñoz-Leal and Mans, 2024
181	*Ornithodoros papillipes* (Birula, 1895)
182	*Ornithodoros parkeri* Cooley, 1936
183	*Ornithodoros pavimentosus* Neumann, 1901
184	*Ornithodoros phacochoerus* Bakkes, de Klerk and Mans, 2018
185	*Ornithodoros porcinus* Walton, 1962
186	*Ornithodoros procaviae* Theodor and Costa, 1960
187	*Ornithodoros rostratus* Aragão, 1911
188	*Ornithodoros rupestris* Trape, Bitam, Renaud and Durand, 2013
189	*Ornithodoros savignyi* (Audouin, 1826)
190	*Ornithodoros sonrai* Sautet and Witkowski, 1943
191	*Ornithodoros sparnus* Kohls and Clifford, 1963
192	*Ornithodoros tartakovskyi* Olenev, 1931
193	*Ornithodoros tholozani* (Laboulbène and Mégnin, 1882)
194	*Ornithodoros transversus* (Banks, 1902)
195	*Ornithodoros turicata* (Dugès, 1876)
196	*Ornithodoros verrucosus* Olenev, Zasukhin and Fenyuk, 1934
197	*Ornithodoros waterbergensis* Bakkes, de Klerk and Mans, 2018
198	*Ornithodoros zumpti* Heisch and Guggisberg, 1953
199	*Otobius lagophilus* Cooley and Kohls, 1940
200	*Otobius megnini* (Dugès, 1883)
201	*Proknekalia peringueyi* (Bedford and Hewitt, 1925)
202	*Proknekalia peusi* (Schulze, 1943)
203	*Proknekalia vansomereni* (Keirans, Hoogstraal and Clifford, 1977)
204	*Reticulinasus batuensis* (Hirst, 1929)
205	*Reticulinasus camicasi* (Sylla, Cornet and Marchand, 1997)
206	*Reticulinasus chiropterphila* (Dhanda and Rajagopalan, 1971)
207	*Reticulinasus faini* (Hoogstraal, 1960)
208	*Reticulinasus hadiae* (Klompen, Keirans and Durden, 1995)
209	*Reticulinasus madagascariensis* (Hoogstraal, 1962)
210	*Reticulinasus multisetosus* (Klompen, Keirans and Durden, 1995)
211	*Reticulinasus papuensis* (Klompen, Keirans and Durden, 1995)
212	*Reticulinasus piriformis* (Warburton, 1918)
213	*Reticulinasus rennellensis* (Clifford and Sonenshine, 1962)
214	*Reticulinasus salahi* (Hoogstraal, 1953)
215	*Reticulinasus solomonis* (Dumbleton, 1959)
216	*Secretargas echinops* (Hoogstraal, Uilenberg and Blanc, 1967)
217	*Secretargas hoogstraali* (Morel and Vassiliades, 1965)
218	*Secretargas transgariepinus* (White, 1846)
219	*Subparmatus marinkellei* (Kohls, Clifford and Jones, 1969)
220	*Subparmatus mormoops* (Kohls, Clifford and Jones, 1969)
221	*Subparmatus viguerasi* (Cooley and Kohls, 1941)

Genera and species are listed alphabetically, and fossil species are marked with an asterisk (*)

## The family Ixodidae

The family Ixodidae comprises 20 genera and 800 species. Table 3 lists the valid ixodid tick species. This list includes 786 ixodid tick species from Robbins et al. [20], excluding *Ixodes inopinatus* Estrada-Peña, Nava, and Petney, 2014 and *Rhipicephalus hibericus* Millán, Rodríguez-Pastor, and Estrada-Peña, 2024 and adding the following 16 species described or reinstated thereafter: *Amblyomma arawakan* Chitimia-Dobler and Martins, 2025, *Amblyomma formosanum* Schulze, 1933 (reinstated), *Amblyomma pakhtunensis* Ali, Chitimia-Dobler and Mans, 2024, *Dermacentor pseudotamokensis* Hornok, 2025, *Haemaphysalis eleonorae* Chitimia-Dobler, Mans and Saratsis, 2024, *Haemaphysalis mariae* Apanaskevich, 2024, *Haemaphysalis vasilisae* Apanaskevich, 2025, *Ixodes abramovin* Apanaskevich, 2024, *Ixodes algericus* Keskin, Aftisse and Apanaskevich, 2024, *Ixodes ampullaceus* Warburton, 1933 (reinstcated), *Ixodes colboi* Apanaskevich and Hall, 2025, *Ixodes hyracis* Apanaskevich, Drew and Pienaar, 2025, *Ixodes lanigeri* Hornok, 2024, *Ixodes paragibbosus* Hornok and Kontschán, 2025, *Ixodes tatei* Arthur, 1959 (reinstated), and *Ixodes zacateco* Apanaskevich and Bunn, 2025 [41–53]. The newly described species *Amblyomma kappa* [54] was recently synonymized with *Amblyomma formosanum* Schulze, 1933 [55] and is, therefore, not included here. *Amblyomma formosanum* is, in turn, listed here as a valid species. The species *I. inopinatus* [56, 57] is regarded here as a synonym of *Ixodes ricinus* (Linnaeus, 1758). Their mitochondrial genome identities (> 99% pairwise sequence similarity for the whole mitochondrial genome) within the *I. ricinus* species complex are well within accepted species variation ranges [16], suggesting ecotypes rather than separate species. Similarly, the recently described *R. hibericus* [58] is considered here a junior synonym of *Rhipicephalus sanguineus* (Latreille, 1806). Some males of *R. sanguineus* from certain European countries exhibit relatively larger spiracular plate tails, but these are intraspecific morphological variations [59, 60]. In fact, these males are essentially genetically indistinguishable from *R. sanguineus* [61, 62]. These species are, therefore, not included in the current list.

**Table 3:** List of valid species of hard ticks (family Ixodidae)

No	Species
1	*Africaniella orlovi* (Kolonin, 1992)
2	*Africaniella transversale* (Lucas, 1845)
3	*Alloceraea aponommoides* (Warburton, 1913)
4	*Alloceraea colasbelcouri* (Santos Dias, 1958)
5	*Alloceraea cretacea* (Chitimia-Dobler, Pfeffer and Dunlop, 2018)*
6	*Alloceraea inermis* (Birula, 1895)
7	*Alloceraea kitaokai* (Hoogstraal, 1969)
8	*Alloceraea kolonini* (Du, Sun, Xu and Shao, 2018)
9	*Alloceraea primitiva* (Teng, 1982)
10	*Amblyomma albolimbatum* Neumann, 1907
11	*Amblyomma albopictum* Neumann, 1899
12	*Amblyomma americanum* (Linnaeus, 1758)
13	*Amblyomma anicornuta* Apanaskevich and Apanaskevich, 2018
14	*Amblyomma antillorum* Kohls, 1969
15	*Amblyomma arawakan* Chitimia-Dobler and Martins, 2025
16	*Amblyomma arcanum* Karsch, 1879
17	*Amblyomma argentinae* Neumann, 1905
18	*Amblyomma astrion* Dönitz, 1909
19	*Amblyomma aureolatum* (Pallas, 1772)
20	*Amblyomma auricularium* (Conil, 1878)
21	*Amblyomma australiense* Neumann, 1905
22	*Amblyomma babirussae* Schulze, 1933
23	*Amblyomma beaurepairei* Vogelsang and Santos Dias, 1953
24	*Amblyomma birmitum* Chitimia-Dobler, Araujo, Ruthensteiner, Pfeffer and Dunlop, 2017*
25	*Amblyomma boeroi* Nava, Mangold, Mastropaolo, Venzal, Oscherov and Guglielmone, 2009
26	*Amblyomma boulengeri* Hirst and Hirst, 1910
27	*Amblyomma brasiliense* Aragão, 1908
28	*Amblyomma breviscutatum* Neumann, 1899
29	*Amblyomma cajennense* (Fabricius, 1787)
30	*Amblyomma calabyi* Roberts, 1963
31	*Amblyomma calcaratum* Neumann, 1899
32	*Amblyomma chabaudi* Rageau, 1964
33	*Amblyomma clypeolatum* Neumann, 1899
34	*Amblyomma coelebs* Neumann, 1899
35	*Amblyomma cohaerens* Dönitz, 1909
36	*Amblyomma compressum* (Macalister, 1872)
37	*Amblyomma cordiferum* Neumann, 1899
38	*Amblyomma crassipes* (Neumann, 1901)
39	*Amblyomma crassum* Robinson, 1926
40	*Amblyomma crenatum* Neumann, 1899
41	*Amblyomma cruciferum* Neumann, 1901
42	*Amblyomma darwini* Hirst and Hirst, 1910
43	*Amblyomma dissimile* Koch, 1844
44	*Amblyomma dubitatum* Neumann, 1899
45	*Amblyomma eburneum* Gerstäcker, 1873
46	*Amblyomma echidnae* Roberts, 1953
47	*Amblyomma exornatum* Koch, 1844
48	*Amblyomma extraoculatum* Neumann, 1899
49	*Amblyomma falsomarmoreum* Tonelli Rondelli, 1935
50	*Amblyomma fimbriatum* Koch, 1844
51	*Amblyomma flavomaculatum* (Lucas, 1846)
52	*Amblyomma formosanum* Schulze, 1933
53	*Amblyomma fulvum* Neumann, 1899
54	*Amblyomma fuscolineatum* (Lucas, 1847)
55	*Amblyomma fuscum* Neumann, 1907
56	*Amblyomma geayi* Neumann, 1899
57	*Amblyomma gemma* Dönitz, 1909
58	*Amblyomma geochelone* Durden, Keirans and Smith, 2002
59	*Amblyomma geoemydae* (Cantor, 1847)
60	*Amblyomma gervaisi* (Lucas, 1847)
61	*Amblyomma glauerti* Keirans, King and Sharrad, 1994
62	*Amblyomma goeldii* Neumann, 1899
63	*Amblyomma hadanii* Nava, Mastropaolo, Mangold, Venzal and Guglielmone, 2014
64	*Amblyomma hainanense* Teng, 1981
65	*Amblyomma hebraeum* Koch, 1844
66	*Amblyomma helvolum* Koch, 1844
67	*Amblyomma hirtum* Neumann, 1906
68	*Amblyomma humerale* Koch, 1844
69	*Amblyomma incisum* Neumann, 1906
70	*Amblyomma inopinatum* (Santos Dias, 1989)
71	*Amblyomma inornatum* (Banks, 1909)
72	*Amblyomma integrum* Karsch, 1879
73	*Amblyomma interandinum* Beati, Nava and Cáceres, 2014
74	*Amblyomma javanense* (Supino, 1897)
75	*Amblyomma komodoense* (Oudemans, 1928)
76	*Amblyomma kraneveldi* (Anastos, 1956)
77	*Amblyomma latepunctatum* Tonelli Rondelli, 1939
78	*Amblyomma latum* Koch, 1844
79	*Amblyomma lepidum* Dönitz, 1909
80	*Amblyomma limbatum* Neumann, 1899
81	*Amblyomma loculosum* Neumann, 1907
82	*Amblyomma longirostre* (Koch, 1844)
83	*Amblyomma macfarlandi* Keirans, Hoogstraal and Clifford, 1973
84	*Amblyomma macropi* Roberts, 1953
85	*Amblyomma maculatum* Koch, 1844
86	*Amblyomma marmoreum* Koch, 1844
87	*Amblyomma mixtum* Koch, 1844
88	*Amblyomma monteiroae* Soares, Labruna and Martins, 2023
89	*Amblyomma moreliae* (Koch, 1867)
90	*Amblyomma moyi* Roberts, 1953
91	*Amblyomma multipunctum* Neumann, 1899
92	*Amblyomma naponense* (Packard, 1869)
93	*Amblyomma neumanni* Ribaga, 1902
94	*Amblyomma nitidum* Hirst and Hirst, 1910
95	*Amblyomma nodosum* Neumann, 1899
96	*Amblyomma nuttalli* Dönitz, 1909
97	*Amblyomma oblongoguttatum* Koch, 1844
98	*Amblyomma ovale* Koch, 1844
99	*Amblyomma pacae* Aragão, 1911
100	*Amblyomma pakhtunensis* Ali, Chitimia-Dobler and Mans, 2024
101	*Amblyomma parkeri* Fonseca and Aragão, 1952
102	*Amblyomma parvitarsum* Neumann, 1901
103	*Amblyomma parvum* Aragão, 1908
104	*Amblyomma patinoi* Labruna, Nava and Beati, 2014
105	*Amblyomma pattoni* (Neumann, 1910)
106	*Amblyomma paulopunctatum* Neumann, 1899
107	*Amblyomma pecarium* Dunn, 1933
108	*Amblyomma personatum* Neumann, 1901
109	*Amblyomma pictum* Neumann, 1906
110	*Amblyomma pilosum* Neumann, 1899
111	*Amblyomma pomposum* Dönitz, 1909
112	*Amblyomma postoculatum* Neumann, 1899
113	*Amblyomma pseudoconcolor* Aragão, 1908
114	*Amblyomma pseudoparvum* Guglielmone, Mangold and Keirans, 1990
115	*Amblyomma quadricavum* (Schulze, 1941)
116	*Amblyomma rhinocerotis* (De Geer, 1778)
117	*Amblyomma robinsoni* Warburton, 1927
118	*Amblyomma romarioi* Martins, Luz and Labruna, 2019
119	*Amblyomma romitii* Tonelli Rondelli, 1939
120	*Amblyomma rotundatum* Koch, 1844
121	*Amblyomma sabanerae* Stoll, 1894
122	*Amblyomma scalpturatum* Neumann, 1906
123	*Amblyomma sculptum* Berlese, 1888
124	*Amblyomma scutatum* Neumann, 1899
125	*Amblyomma soembawense* (Anastos, 1956)
126	*Amblyomma sparsum* Neumann, 1899
127	*Amblyomma splendidum* Giebel, 1877
128	*Amblyomma squamosum* Kohls, 1953
129	*Amblyomma supinoi* Neumann, 1905
130	*Amblyomma sylvaticum* (De Geer, 1778)
131	*Amblyomma tapirellum* Dunn, 1933
132	*Amblyomma tenellum* Koch, 1844
133	*Amblyomma testudinarium* Koch, 1844
134	*Amblyomma tholloni* Neumann, 1899
135	*Amblyomma tigrinum* Koch, 1844
136	*Amblyomma tonelliae* Nava, Beati and Labruna, 2014
137	*Amblyomma torrei* Pérez Vigueras, 1934
138	*Amblyomma triguttatum* Koch, 1844
139	*Amblyomma trimaculatum* (Lucas, 1878)
140	*Amblyomma triste* Koch, 1844
141	*Amblyomma tuberculatum* Marx, 1894
142	*Amblyomma usingeri* Keirans, Hoogstraal and Clifford, 1973
143	*Amblyomma varanense* (Supino, 1897)
144	*Amblyomma variegatum* (Fabricius, 1798)
145	*Amblyomma varium* Koch, 1844
146	*Amblyomma vikirri* Keirans, Bull and Duffield, 1996
147	*Amblyomma williamsi* Banks, 1924
148	*Amblyomma yucumense* Krawczak, Martins and Labruna, 2015
149	*Anomalohimalaya cricetuli* Teng and Huang, 1981
150	*Anomalohimalaya lamai* Hoogstraal, Kaiser and Mitchell, 1970
151	*Anomalohimalaya lotozkyi* Filippova and Panova, 1978
152	*Archaecroton kaufmani* Chitimia-Dobler, Mans and Dunlop, 2023*
153	*Archaeocroton sphenodonti* (Dumbleton, 1943)
154	*Bothriocroton auruginans* (Schulze, 1936)
155	*Bothriocroton concolor* (Neumann, 1899)
156	*Bothriocroton glebopalma* (Keirans, King and Sharrad, 1994)
157	*Bothriocroton hydrosauri* (Denny, 1843)
158	*Bothriocroton muelleri* Chitimia-Dobler, Mans and Dunlop, 2023*
159	*Bothriocroton oudemansi* (Neumann, 1910)
160	*Bothriocroton tachyglossi* (Roberts, 1953)
161	*Bothriocroton undatum* (Fabricius, 1775)
162	*Compluriscutula vetulum* Poinar and Buckley, 2008*
163	*Cornupalpatum burmanicum* Poinar and Brown, 2003*
164	*Cosmiomma hippopotamensis* (Denny, 1843)
165	*Cryptocroton papuanum* (Hirst, 1914)
166	*Dermacentor albipictus* (Packard, 1869)
167	*Dermacentor andersoni* Stiles, 1908
168	*Dermacentor asper* Arthur, 1960
169	*Dermacentor auratus* Supino, 1897
170	*Dermacentor bellulus* (Schulze, 1935)
171	*Dermacentor circumguttatus* Neumann, 1897
172	*Dermacentor compactus* Neumann, 1901
173	*Dermacentor confragus* (Schulze, 1933)
174	*Dermacentor dispar* Cooley, 1937
175	*Dermacentor dissimilis* Cooley, 1947
176	*Dermacentor everestianus* Hirst, 1926
177	*Dermacentor falsosteini* Apanaskevich, Apanaskevich and Nooma, 2021
178	*Dermacentor filippovae* Apanaskevich and Apanaskevich, 2015
179	*Dermacentor halli* McIntosh, 1931
180	*Dermacentor hunteri* Bishopp, 1912
181	*Dermacentor imitans* Warburton, 1933
182	*Dermacentor kamshadalus* Neumann, 1908
183	*Dermacentor laothaiensis* Apanaskevich, Chaloemthanetphong and Vongphayloth, 2019
184	*Dermacentor latus* Cooley, 1937
185	*Dermacentor limbooliati* Apanaskevich and Apanaskevich, 2015
186	*Dermacentor marginatus* (Sulzer, 1776)
187	*Dermacentor montanus* Filippova and Panova, 1974
188	*Dermacentor nitens* Neumann, 1897
189	*Dermacentor niveus* Neumann, 1897
190	*Dermacentor nuttalli* Olenev, 1929
191	*Dermacentor occidentalis* Marx, 1892
192	*Dermacentor panamensis* Apanaskevich and Bermúdez, 2013
193	*Dermacentor parumapertus* Neumann, 1901
194	*Dermacentor pasteuri* Apanaskevich, Vongphayloth, Jeangkhwoa and Chaloemthanetphong, 2020
195	*Dermacentor pavlovskyi* Olenev, 1927
196	*Dermacentor pomerantzevi* Serdjukova, 1951
197	*Dermacentor pseudotamokensis* Hornok, 2025
198	*Dermacentor pseudocompactus* Apanaskevich and Apanaskevich, 2016
199	*Dermacentor raskemensis* Pomerantzev, 1946
200	*Dermacentor reticulatus* (Fabricius, 1794)
201	*Dermacentor rhinocerinus* (Denny, 1843)
202	*Dermacentor silvarum* Olenev, 1931
203	*Dermacentor similis* Lado, Glon and Klompen, 2021
204	*Dermacentor sinicus* Schulze, 1931
205	*Dermacentor steini* (Schulze, 1933)
206	*Dermacentor taiwanensis* Sugimoto, 1935
207	*Dermacentor tamokensis* Apanaskevich and Apanaskevich, 2016
208	*Dermacentor tricuspis* (Schulze, 1933)
209	*Dermacentor ushakovae* Filippova and Panova, 1987
210	*Dermacentor variabilis* (Say, 1821)
211	*Haemaphysalis aborensis* Warburton, 1913
212	*Haemaphysalis aciculifer* Warburton, 1913
213	*Haemaphysalis aculeata* Lavarra, 1904
214	*Haemaphysalis adleri* Feldman-Muhsam, 1951
215	*Haemaphysalis anomala* Warburton, 1913
216	*Haemaphysalis anomaloceraea* Teng, 1984
217	*Haemaphysalis anoplos* Hoogstraal, Uilenberg and Klein, 1967
218	*Haemaphysalis asiatica* (Supino, 1897)
219	*Haemaphysalis atheruri* Hoogstraal, Trapido and Kohls, 1965
220	*Haemaphysalis bancrofti* Nuttall and Warburton, 1915
221	*Haemaphysalis bandicota* Hoogstraal and Kohls, 1965
222	*Haemaphysalis bartelsi* Schulze, 1938
223	*Haemaphysalis bequaerti* Hoogstraal, 1956
224	*Haemaphysalis birmaniae* Supino, 1897
225	*Haemaphysalis bispinosa* Neumann, 1897
226	*Haemaphysalis bochkovi* Apanaskevich and Tomlinson, 2019
227	*Haemaphysalis borneata* Hoogstraal, 1971
228	*Haemaphysalis bremneri* Roberts, 1963
229	*Haemaphysalis burkinae* Apanaskevich and Tomlinson, 2019
230	*Haemaphysalis calcarata* Neumann, 1902
231	*Haemaphysalis calva* Nuttall and Warburton, 1915
232	*Haemaphysalis camicasi* Tomlinson and Apanaskevich, 2019
233	*Haemaphysalis campanulata* Warburton, 1908
234	*Haemaphysalis canestrinii* (Supino, 1897)
235	*Haemaphysalis capricornis* Hoogstraal, 1966
236	*Haemaphysalis caucasica* Olenev, 1928
237	*Haemaphysalis celebensis* Hoogstraal, Trapido and Kohls, 1965
238	*Haemaphysalis chordeilis* (Packard, 1869)
239	*Haemaphysalis cinnabarina* Koch, 1844
240	*Haemaphysalis colesbergensis* Apanaskevich and Horak, 2008
241	*Haemaphysalis concinna* Koch, 1844
242	*Haemaphysalis cooleyi* Bedford, 1929
243	*Haemaphysalis cornigera* Neumann, 1897
244	*Haemaphysalis cornupunctata* Hoogstraal and Varma, 1962
245	*Haemaphysalis cuspidata* Warburton, 1910
246	*Haemaphysalis dangi* Phan Trong, 1977
247	*Haemaphysalis danieli* Černý and Hoogstraal, 1977
248	*Haemaphysalis darjeeling* Hoogstraal and Dhanda, 1970
249	*Haemaphysalis davisi* Hoogstraal, Dhanda and Bhat, 1970
250	*Haemaphysalis demidovae* Emel’yanova, 1978
251	*Haemaphysalis dentipalpis* Warburton and Nuttall, 1909
252	*Haemaphysalis doenitzi* Warburton and Nuttall, 1909
253	*Haemaphysalis eleonorae* Chitimia-Dobler, Mans and Saratsis, 2024
254	*Haemaphysalis elliptica* (Koch, 1844)
255	*Haemaphysalis elongata* Neumann, 1897
256	*Haemaphysalis erinacei* Pavesi, 1884
257	*Haemaphysalis eupleres* Hoogstraal, Kohls and Trapido, 1965
258	*Haemaphysalis filippovae* Bolotin, 1979
259	*Haemaphysalis flava* Neumann, 1897
260	*Haemaphysalis formosensis* Neumann, 1913
261	*Haemaphysalis fossae* Hoogstraal, 1953
262	*Haemaphysalis fujisana* Kitaoka, 1970
263	*Haemaphysalis galidiae* Apanaskevich and Goodman, 2020
264	*Haemaphysalis garhwalensis* Dhanda and Bhat, 1968
265	*Haemaphysalis goral* Hoogstraal, 1970
266	*Haemaphysalis grochovskajae* Kolonin, 1992
267	*Haemaphysalis heinrichi* Schulze, 1939
268	*Haemaphysalis hirsuta* Hoogstraal, Trapido and Kohls, 1966
269	*Haemaphysalis hispanica* Gill Collado, 1938
270	*Haemaphysalis hoodi* Warburton and Nuttall, 1909
271	*Haemaphysalis hoogstraali* Kohls, 1950
272	*Haemaphysalis horaki* Apanaskevich and Tomlinson, 2019
273	*Haemaphysalis houyi* Nuttall and Warburton, 1915
274	*Haemaphysalis howletti* Warburton, 1913
275	*Haemaphysalis humerosa* Warburton and Nuttall, 1909
276	*Haemaphysalis hylobatis* Schulze, 1933
277	*Haemaphysalis hyracophila* Hoogstraal, Walker and Neitz, 1971
278	*Haemaphysalis hystricis* Supino, 1897
279	*Haemaphysalis indica* Warburton, 1910
280	*Haemaphysalis indoflava* Dhanda and Bhat, 1968
281	*Haemaphysalis intermedia* Warburton and Nuttall, 1909
282	*Haemaphysalis japonica* Warburton, 1908
283	*Haemaphysalis juxtakochi* Cooley, 1946
284	*Haemaphysalis kadarsani* Hoogstraal and Wassef, 1977
285	*Haemaphysalis kashmirensis* Hoogstraal and Varma, 1962
286	*Haemaphysalis kinneari* Warburton, 1913
287	*Haemaphysalis knobigera* Prakasan and Ramani, 2007
288	*Haemaphysalis koningsbergeri* Warburton and Nuttall, 1909
289	*Haemaphysalis kopetdaghica* Kerbabaev, 1962
290	*Haemaphysalis kumaonensis* Geevarghese and Mishra, 2011
291	*Haemaphysalis kutchensis* Hoogstraal and Trapido, 1963
292	*Haemaphysalis kyasanurensis* Trapido, Hoogstraal and Rajagopalan, 1964
293	*Haemaphysalis lagostrophi* Roberts, 1963
294	*Haemaphysalis lagrangei* Larrousse, 1925
295	*Haemaphysalis laocayensis* Phan Trong, 1977
296	*Haemaphysalis latitudinis* Apanaskevich and Tomlinson, 2020
297	*Haemaphysalis leachi* (Audouin, 1826)
298	*Haemaphysalis lemuris* Hoogstraal, 1953
299	*Haemaphysalis leporispalustris* (Packard, 1869)
300	*Haemaphysalis lobachovi* Kolonin, 1995
301	*Haemaphysalis longicornis* Neumann, 1901
302	*Haemaphysalis luzonensis* Hoogstraal and Parrish, 1968
303	*Haemaphysalis madagascariensis* Colas-Belcour and Millot, 1948
304	*Haemaphysalis mageshimaensis* Saito and Hoogstraal, 1973
305	*Haemaphysalis mariae* Apanaskevich, 2024
306	*Haemaphysalis megalaimae* Rajagopalan, 1963
307	*Haemaphysalis megaspinosa* Saito, 1969
308	*Haemaphysalis menglaensis* Pang, Chen and Xiang, 1982
309	*Haemaphysalis minuta* Kohls, 1950
310	*Haemaphysalis mjoebergi* Warburton, 1926
311	*Haemaphysalis montgomeryi* Nuttall, 1912
312	*Haemaphysalis moreli* Camicas, Hoogstraal and El Kammah, 1972
313	*Haemaphysalis moschisuga* Teng, 1980
314	*Haemaphysalis muhsamae* Santos Dias, 1954
315	*Haemaphysalis nadchatrami* Hoogstraal, Trapido and Kohls, 1965
316	*Haemaphysalis nepalensis* Hoogstraal, 1962
317	*Haemaphysalis nesomys* Hoogstraal, Uilenberg and Klein, 1966
318	*Haemaphysalis norvali* Hoogstraal and Wassef, 1983
319	*Haemaphysalis novaeguineae* Hirst, 1914
320	*Haemaphysalis obesa* Larrousse, 1925
321	*Haemaphysalis obtusa* Dönitz, 1910
322	*Haemaphysalis oliveri* Apanaskevich and Horak, 2008
323	*Haemaphysalis orientalis* Nuttall and Warburton, 1915
324	*Haemaphysalis ornithophila* Hoogstraal and Kohls, 1959
325	*Haemaphysalis palawanensis* Kohls, 1950
326	*Haemaphysalis papuana* Thorell, 1883
327	*Haemaphysalis paraleachi* Camicas, Hoogstraal and El Kammah, 1983
328	*Haemaphysalis paraturturis* Hoogstraal, Trapido and Rebello, 1963
329	*Haemaphysalis parmata* Neumann, 1905
330	*Haemaphysalis parva* (Neumann, 1897)
331	*Haemaphysalis pavlovskyi* Pospelova-Shtrom, 1935
332	*Haemaphysalis pedetes* Hoogstraal, 1972
333	*Haemaphysalis pentalagi* Pospelova-Shtrom, 1935
334	*Haemaphysalis petrogalis* Roberts, 1970
335	*Haemaphysalis phasiana* Saito, Hoogstraal and Wassef, 1974
336	*Haemaphysalis pospelovashtromae* Hoogstraal, 1966
337	*Haemaphysalis princeps* Tomlinson and Apanaskevich, 2019
338	*Haemaphysalis psalistos* Hoogstraal, Kohls and Parrish, 1967
339	*Haemaphysalis punctaleachi* Camicas, Hoogstraal and El Kammah, 1973
340	*Haemaphysalis punctata* Canestrini and Fanzago, 1878
341	*Haemaphysalis qinghaiensis* Teng, 1980
342	*Haemaphysalis quadriaculeata* Kolonin, 1992
343	*Haemaphysalis ramachandrai* Dhanda, Hoogstraal and Bhat, 1970
344	*Haemaphysalis ratti* Kohls, 1948
345	*Haemaphysalis renschi* Schulze, 1933
346	*Haemaphysalis roubaudi* Toumanoff, 1940
347	*Haemaphysalis rugosa* Santos Dias, 1956
348	*Haemaphysalis rusae* Kohls, 1950
349	*Haemaphysalis sambar* Hoogstraal, 1971
350	*Haemaphysalis sciuri* Kohls, 1950
351	*Haemaphysalis semermis* Neumann, 1901
352	*Haemaphysalis setosa* Apanaskevich and Tomlinson, 2020
353	*Haemaphysalis shimoga* Trapido and Hoogstraal, 1964
354	*Haemaphysalis silacea* Robinson, 1912
355	*Haemaphysalis silvafelis* Hoogstraal and Trapido, 1963
356	*Haemaphysalis simplex* Neumann, 1897
357	*Haemaphysalis simplicima* Hoogstraal and Wassef, 1979
358	*Haemaphysalis sinensis* Zhang, 1981
359	*Haemaphysalis spinigera* Neumann, 1897
360	*Haemaphysalis spinulosa* Neumann, 1906
361	*Haemaphysalis subelongata* Hoogstraal, 1953
362	*Haemaphysalis subterra* Hoogstraal, El Kammah and Camicas, 1992
363	*Haemaphysalis sulcata* Canestrini and Fanzago, 1878
364	*Haemaphysalis sumatraensis* Hoogstraal, El Kammah, Kadarsan and Anastos, 1971
365	*Haemaphysalis sundrai* Sharif, 1928
366	*Haemaphysalis suntzovi* Kolonin, 1993
367	*Haemaphysalis susphilippensis* Hoogstraal, Kohls and Parrish, 1968
368	*Haemaphysalis taiwana* Sugimoto, 1936
369	*Haemaphysalis tauffliebi* Morel, 1965
370	*Haemaphysalis tibetensis* Hoogstraal, 1965
371	*Haemaphysalis tiptoni* Hoogstraal, 1953
372	*Haemaphysalis toxopei* Warburton, 1927
373	*Haemaphysalis traguli* Oudemans, 1928
374	*Haemaphysalis traubi* Kohls, 1955
375	*Haemaphysalis turturis* Nuttall and Warburton, 1915
376	*Haemaphysalis vasilisae* Apanaskevich, 2025
377	*Haemaphysalis verticalis* Itagaki, Noda and Yamaguchi, 1944
378	*Haemaphysalis vidua* Warburton and Nuttall, 1909
379	*Haemaphysalis walkerae* Apanaskevich and Tomlinson, 2019
380	*Haemaphysalis warburtoni* Nuttall, 1912
381	*Haemaphysalis wellingtoni* Nuttall and Warburton, 1908
382	*Haemaphysalis yeni* Toumanoff, 1944
383	*Haemaphysalis zumpti* Hoogstraal and El Kammah, 1974
384	*Hyalomma aegyptium* (Linnaeus, 1758)
385	*Hyalomma albiparmatum* Schulze, 1920
386	*Hyalomma anatolicum* Koch, 1844
387	*Hyalomma arabica* Pegram, Hoogstraal and Wassef, 1982
388	*Hyalomma asiaticum* Schulze and Schlottke, 1929
389	*Hyalomma brevipunctatum* Sharif, 1928
390	*Hyalomma dromedarii* Koch, 1844
391	*Hyalomma excavatum* Koch, 1844
392	*Hyalomma franchinii* Tonelli Rondelli, 1932
393	*Hyalomma glabrum* Delpy, 1949
394	*Hyalomma hussaini* Sharif, 1928
395	*Hyalomma hystricis* Dhanda and Raja, 1974
396	*Hyalomma impeltatum* Schulze and Schlottke, 1929
397	*Hyalomma impressum* Koch, 1844
398	*Hyalomma isaaci* Sharif, 1928
399	*Hyalomma kumari* Sharif, 1928
400	*Hyalomma lusitanicum* Koch, 1844
401	*Hyalomma marginatum* Koch, 1844
402	*Hyalomma nitidum* Schulze, 1920
403	*Hyalomma punt* Hoogstraal, Kaiser and Pedersen, 1969
404	*Hyalomma rhipicephaloides* Neumann, 1901
405	*Hyalomma rufipes* Koch, 1844
406	*Hyalomma schulzei* Olenev, 1931
407	*Hyalomma scupense* Schulze, 1919
408	*Hyalomma somalicum* Tonelli Rondelli, 1935
409	*Hyalomma truncatum* Koch, 1844
410	*Hyalomma turanicum* Pomerantzev, 1946
411	*Ixodes abramovin* Apanaskevich, 2024
412	*Ixodes abrocomae* Lahille, 1916
413	*Ixodes acer* Apanaskevich and Schenk, 2020
414	*Ixodes acuminatus* Neumann, 1901
415	*Ixodes acutitarsus* (Karsch, 1880)
416	*Ixodes affinis* Neumann, 1899
417	*Ixodes albignaci* Uilenberg and Hoogstraal, 1969
418	*Ixodes alluaudi* Neumann, 1913
419	*Ixodes algericus* Keskin, Aftisse and Apanaskevich, 2024
420	*Ixodes amarali* Fonseca, 1935
421	*Ixodes ambohitantelensis* Englert, Goodman, Apanaskevich, 2023
422	*Ixodes amersoni* Kohls, 1966
423	*Ixodes ampullaceus* Warburton, 1933
424	*Ixodes anatis* Chilton, 1904
425	*Ixodes andinus* Kohls, 1956
426	*Ixodes angustus* Neumann, 1899
427	*Ixodes antechini* Roberts, 1960
428	*Ixodes antiquorum* Chitimia-Dobler, Mans and Dunlop, 2022*
429	*Ixodes apronophorus* Schulze, 1924
430	*Ixodes arabukiensis* Arthur, 1959
431	*Ixodes arboricola* Schulze and Schlottke, 1929
432	*Ixodes arebiensis* Arthur, 1956
433	*Ixodes ariadnae* Hornok, 2014
434	*Ixodes asanumai* Kitaoka, 1973
435	*Ixodes aulacodi* Arthur, 1956
436	*Ixodes auriculaelongae* Arthur, 1958
437	*Ixodes auritulus* Neumann, 1904
438	*Ixodes australiensis* Neumann, 1904
439	*Ixodes baergi* Cooley and Kohls, 1942
440	*Ixodes bakeri* Arthur and Clifford, 1961
441	*Ixodes banksi* Bishopp, 1911
442	*Ixodes barkeri* Barker, 2019
443	*Ixodes bedfordi* Arthur, 1959
444	*Ixodes bequaerti* Cooley and Kohls, 1945
445	*Ixodes berlesei* Birula, 1895
446	*Ixodes bivari* Santos Dias, 1990
447	*Ixodes bocatorensis* Apanaskevich and Bermúdez, 2017
448	*Ixodes boliviensis* Neumann, 1904
449	*Ixodes brevisetosus* Apanaskevich, 2022
450	*Ixodes brewsterae* Keirans, Clifford and Walker, 1982
451	*Ixodes browningi* Arthur, 1956
452	*Ixodes brumpti* Morel, 1965
453	*Ixodes brunneus* Koch, 1844
454	*Ixodes calcarhebes* Arthur and Zulu, 1980
455	*Ixodes caledonicus* Nuttall, 1910
456	*Ixodes canisuga* Johnston, 1849
457	*Ixodes capromydis* Černý, 1966
458	*Ixodes catarinensis* Onofrio and Labruna, 2020
459	*Ixodes catherinei* Keirans, Clifford and Walker, 1982
460	*Ixodes cavipalpus* Nuttall and Warburton, 1908
461	*Ixodes ceylonensis* Kohls, 1950
462	*Ixodes chacoensis* Nava, Beati, Venzal and Guglielmone, 2023
463	*Ixodes chilensis* Kohls, 1956
464	*Ixodes colasbelcouri* Arthur, 1957
465	*Ixodes colboi* Apanaskevich and Hall, 2025
466	*Ixodes collaris* Hornok, 2016
467	*Ixodes collocaliae* Schulze, 1937
468	*Ixodes columnae* Takada and Fujita, 1992
469	*Ixodes conepati* Cooley and Kohls, 1943
470	*Ixodes confusus* Roberts, 1960
471	*Ixodes contrarius* Apanaskevich, 2022
472	*Ixodes cookei* Packard, 1869
473	*Ixodes cooleyi* Aragão and Fonseca, 1951
474	*Ixodes copei* Wilson, 1980
475	*Ixodes cordifer* Neumann, 1908
476	*Ixodes cornuae* Arthur, 1960
477	*Ixodes cornuatus* Roberts, 1960
478	*Ixodes cornutus* Lotozky, 1956
479	*Ixodes corwini* Keirans, Clifford and Walker, 1982
480	*Ixodes crenulatus* Koch, 1844
481	*Ixodes cuernavacensis* Kohls and Clifford, 1966
482	*Ixodes cumulatimpunctatus* Schulze, 1943
483	*Ixodes dampfi* Cooley, 1943
484	*Ixodes daveyi* Nuttall, 1913
485	*Ixodes dawesi* Arthur, 1956
486	*Ixodes dendrolagi* Wilson, 1967
487	*Ixodes dentatus* Marx, 1899
488	*Ixodes dicei* Keirans and Ajohda, 2003
489	*Ixodes diomedeae* Arthur, 1958
490	*Ixodes diversifossus* Neumann, 1899
491	*Ixodes djaronensis* Neumann, 1907
492	*Ixodes domerguei* Uilenberg and Hoogstraal, 1965
493	*Ixodes downsi* Kohls, 1957
494	*Ixodes drakensbergensis* Clifford, Theiler and Baker, 1975
495	*Ixodes eadsi* Kohls and Clifford, 1964
496	*Ixodes eastoni* Keirans and Clifford, 1983
497	*Ixodes eichhorni* Nuttall, 1916
498	*Ixodes eldaricus* Dzhaparidze, 1950
499	*Ixodes elongatus* Bedford, 1929
500	*Ixodes eudyptidis* Maskell, 1885
501	*Ixodes euplecti* Arthur, 1958
502	*Ixodes evansi* Arthur, 1956
503	*Ixodes fecialis* Warburton and Nuttall, 1909
504	*Ixodes festai* Tonelli Rondelli, 1926
505	*Ixodes filippovae* Černý, 1961
506	*Ixodes fossulatus* Neumann, 1899
507	*Ixodes frontalis* (Panzer, 1798)
508	*Ixodes fujitai* Hornok and Takano, 2023
509	*Ixodes fuliginosus* Hornok and Takano, 2023
510	*Ixodes fuscipes* Koch, 1844
511	*Ixodes fynbosensis* Apanaskevich, Horak, Matthee and Matthee, 2011
512	*Ixodes galapagoensis* Clifford and Hoogstraal, 1980
513	*Ixodes ghilarovi* Filippova and Panova, 1988
514	*Ixodes gibbosus* Nuttall, 1916
515	*Ixodes giluwensis* Apanaskevich and Schenk, 2020
516	*Ixodes goliath* Apanaskevich and Lemon, 2018
517	*Ixodes granulatus* Supino, 1897
518	*Ixodes gregsoni* Lindquist, Wu and Redner, 1999
519	*Ixodes guatemalensis* Kohls, 1956
520	*Ixodes guglielmonei* Apanaskevich, 2022
521	*Ixodes hearlei* Gregson, 1941
522	*Ixodes heathi* Kwak, 2018
523	*Ixodes heinrichi* Arthur, 1962
524	*Ixodes hexagonus* Leach, 1815
525	*Ixodes himalayensis* Dhanda and Kulkarni, 1969
526	*Ixodes hirsti* Hassall, 1931
527	*Ixodes holocyclus* Neumann, 1899
528	*Ixodes hoogstraali* Arthur, 1955
529	*Ixodes howelli* Cooley and Kohls, 1938
530	*Ixodes hunanensis* Apanaskevich and Duan, 2022
531	*Ixodes hyatti* Clifford, Hoogstraal and Kohls, 1971
532	*Ixodes hydromyidis* Swan, 1931
533	*Ixodes hyracis* Apanaskevich, Drew and Pienaar, 2025
534	*Ixodes insulae* Apanaskevich and Barker, 2022
535	*Ixodes jacksoni* Hoogstraal, 1967
536	*Ixodes jellisoni* Cooley and Kohls, 1938
537	*Ixodes jonesae* Kohls, Sonenshine and Clifford, 1969
538	*Ixodes kaiseri* Arthur, 1957
539	*Ixodes kandingensis* Guo, Sun, Xu and Durden, 2017
540	*Ixodes kashmiricus* Pomerantzev, 1948
541	*Ixodes kazakstani* Olenev and Sorokoumov, 1934
542	*Ixodes keiransi* Beati, Nava, Venzal and Guglielmone, 2023
543	*Ixodes kerguelenensis* André and Colas-Belcour, 1942
544	*Ixodes kingi* Bishopp, 1911
545	*Ixodes kohlsi* Arthur, 1955
546	*Ixodes kopsteini* (Oudemans, 1926)
547	*Ixodes kuntzi* Hoogstraal and Kohls, 1965
548	*Ixodes laguri* Olenev, 1929
549	*Ixodes lanigeri* Hornok, 2024
550	*Ixodes laridis* Heath and Palma, 2017
551	*Ixodes lasallei* Méndez Arocha and Ortiz, 1958
552	*Ixodes latus* Arthur, 1958
553	*Ixodes laysanensis* Wilson, 1964
554	*Ixodes lemuris* Arthur, 1958
555	*Ixodes lewisi* Arthur, 1965
556	*Ixodes lividus* Koch, 1844
557	*Ixodes longiscutatus* Boero, 1944
558	*Ixodes loricatus* Neumann, 1899
559	*Ixodes loveridgei* Arthur, 1958
560	*Ixodes luciae* Sénevet, 1940
561	*Ixodes lunatus* Neumann, 1907
562	*Ixodes luxuriosus* Schulze, 1935
563	*Ixodes macfarlanei* Keirans, Clifford and Walker, 1982
564	*Ixodes malayensis* Kohls, 1962
565	*Ixodes marmotae* Cooley and Kohls, 1938
566	*Ixodes marxi* Banks, 1908
567	*Ixodes maslovi* Emel’yanova and Kozlovskaya, 1967
568	*Ixodes matopi* Spickett, Keirans, Norval and Clifford, 1981
569	*Ixodes mexicanus* Cooley and Kohls, 1942
570	*Ixodes microgalei* Apanaskevich, Soarimalala and Goodman, 2013
571	*Ixodes minor* Neumann, 1902
572	*Ixodes minutae* Arthur, 1959
573	*Ixodes mirzai* Apanaskevich and Schenk, 2020
574	*Ixodes mitchelli* Kohls, Clifford and Hoogstraal, 1970
575	*Ixodes mojavensis* Backus and Beati, 2022
576	*Ixodes monospinosus* Saito, 1967
577	*Ixodes montoyanus* Cooley, 1944
578	*Ixodes moralesi* Apanaskevich and Bermúdez, 2022
579	*Ixodes moreli* Arthur, 1957
580	*Ixodes moscharius* Teng, 1982
581	*Ixodes moschiferi* Nemenz, 1968
582	*Ixodes muniensis* Arthur and Burrow, 1957
583	*Ixodes muris* Bishopp and Smith, 1937
584	*Ixodes murreleti* Cooley and Kohls, 1945
585	*Ixodes myospalacis* Teng, 1986
586	*Ixodes myotomys* Clifford and Hoogstraal, 1970
587	*Ixodes myrmecobii* Roberts, 1962
588	*Ixodes nairobiensis* Nuttall, 1916
589	*Ixodes nchisiensis* Arthur, 1958
590	*Ixodes nectomys* Kohls, 1956
591	*Ixodes neitzi* Clifford, Walker and Keirans, 1977
592	*Ixodes nesomys* Uilenberg and Hoogstraal, 1969
593	*Ixodes neuquenensis* Ringuelet, 1947
594	*Ixodes nicolasi* Santos Dias, 1982
595	*Ixodes nipponensis* Kitaoka and Saito, 1967
596	*Ixodes nipponrhinolophi* Hornok and Takano, 2023
597	*Ixodes nitens* Neumann, 1904
598	*Ixodes nuttalli* Lahille, 1913
599	*Ixodes nuttallianus* Schulze, 1930
600	*Ixodes occultus* Pomerantzev, 1946
601	*Ixodes ochotonae* Gregson, 1941
602	*Ixodes okapiae* Arthur, 1956
603	*Ixodes oldi* Nuttall, 1913
604	*Ixodes ornithorhynchi* Lucas, 1846
605	*Ixodes ovatus* Neumann, 1899
606	*Ixodes pacificus* Cooley and Kohls, 1943
607	*Ixodes paragibbosus* Hornok and Kontschán, 2025
608	*Ixodes paranaensis* Barros-Battesti, Arzua, Pichorim and Keirans, 2003
609	*Ixodes pararicinus* Keirans and Clifford, 1985
610	*Ixodes pavlovskyi* Pomerantzev, 1946
611	*Ixodes percavatus* Neumann, 1906
612	*Ixodes peromysci* Auguston, 1940
613	*Ixodes persulcatus* Schulze, 1930
614	*Ixodes petauristae* Warburton, 1933
615	*Ixodes philipi* Keirans and Kohls, 1970
616	*Ixodes pilosus* Koch, 1844
617	*Ixodes planiscutatus* Apanaskevich, 2020
618	*Ixodes pomerantzevi* Serdjukova, 1941
619	*Ixodes pomerantzi* Kohls, 1956
620	*Ixodes priscicollaris* Schulze, 1932
621	*Ixodes procaviae* Arthur and Burrow, 1957
622	*Ixodes prokopjevi* (Emel’yanova, 1979)
623	*Ixodes radfordi* Kohls, 1948
624	*Ixodes rageaui* Arthur, 1958
625	*Ixodes randrianasoloi* Uilenberg and Hoogstraal, 1969
626	*Ixodes rasus* Neumann, 1899
627	*Ixodes redikorzevi* Olenev, 1927
628	*Ixodes rhabdomysae* Arthur, 1959
629	*Ixodes ricinus* (Linnaeus, 1758)
630	*Ixodes rio* Apanaskevich and Labruna, 2022
631	*Ixodes robbinsi* Apanaskevich and Edgy, 2022
632	*Ixodes rothschildi* Nuttall and Warburton, 1911
633	*Ixodes rotundatus* Arthur, 1958
634	*Ixodes rubicundus* Neumann, 1904
635	*Ixodes rubidus* Neumann, 1901
636	*Ixodes rugicollis* Schulze and Schlottke, 1929
637	*Ixodes rugosus* Bishopp, 1911
638	*Ixodes sachalinensis* Filippova, 1971
639	*Ixodes scapularis* Say, 1821
640	*Ixodes schillingsi* Neumann, 1901
641	*Ixodes schulzei* Aragão and Fonseca, 1951
642	*Ixodes sculptus* Neumann, 1904
643	*Ixodes semenovi* Olenev, 1929
644	*Ixodes shahi* Clifford, Hoogstraal and Kohls, 1971
645	*Ixodes siamensis* Kitaoka and Suzuki, 1983
646	*Ixodes sigelos* Keirans, Clifford and Corwin, 1976
647	*Ixodes signatus* Birula, 1895
648	*Ixodes silvanus* Saracho-Bottero, Beati, Venzal, Guglielmone and Nava, 2021
649	*Ixodes simplex* Neumann, 1906
650	*Ixodes sinaloa* Kohls and Clifford, 1966
651	*Ixodes sinensis* Teng, 1977
652	*Ixodes soarimalalae* Apanaskevich and Goodman, 2020
653	*Ixodes soricis* Gregson, 1942
654	*Ixodes spinae* Arthur, 1958
655	*Ixodes spinicoxalis* Neumann, 1899
656	*Ixodes spinipalpis* Hadwen and Nuttall, 1916
657	*Ixodes spinosus* Neumann, 1899
658	*Ixodes steini* Schulze, 1935
659	*Ixodes stellae* Apanaskevich, 2020
660	*Ixodes stilesi* Neumann, 1911
661	*Ixodes stromi* Filippova, 1957
662	*Ixodes subterraneus* Filippova, 1961
663	*Ixodes succineus* Weidner, 1964*
664	*Ixodes taglei* Kohls, 1969
665	*Ixodes tamaulipas* Kohls and Clifford, 1966
666	*Ixodes tancitarius* Cooley and Kohls, 1942
667	*Ixodes tanuki* Saito, 1964
668	*Ixodes tapirus* Kohls, 1956
669	*Ixodes tasmani* Neumann, 1899
670	*Ixodes tatei* Arthur, 1959
671	*Ixodes tecpanensis* Kohls, 1956
672	*Ixodes texanus* Banks, 1909
673	*Ixodes theilerae* Arthur, 1953
674	*Ixodes thomasae* Arthur and Burrow, 1957
675	*Ixodes tinamou* Apanaskevich, 2022
676	*Ixodes tiptoni* Kohls and Clifford, 1962
677	*Ixodes tovari* Cooley, 1945
678	*Ixodes transvaalensis* Clifford and Hoogstraal, 1966
679	*Ixodes trianguliceps* Birula, 1895
680	*Ixodes trichosuri* Roberts, 1960
681	*Ixodes tropicalis* Kohls, 1956
682	*Ixodes turdus* Nakatsudi, 1942
683	*Ixodes ugandanus* Neumann, 1906
684	*Ixodes uilenbergi* Apanaskevich and Goodman, 2020
685	*Ixodes uncus* Apanaskevich and Goodman, 2020
686	*Ixodes unicavatus* Neumann, 1908
687	*Ixodes uriae* White, 1852
688	*Ixodes vanidicus* Schulze, 1943
689	*Ixodes venezuelensis* Kohls, 1953
690	*Ixodes ventalloi* Gil Collado, 1936
691	*Ixodes vespertilionis* Koch, 1844
692	*Ixodes vestitus* Neumann, 1908
693	*Ixodes victoriensis* Nuttall, 1916
694	*Ixodes walkerae* Clifford, Kohls and Hoogstraal, 1968
695	*Ixodes werneri* Kohls, 1950
696	*Ixodes woodi* Bishopp, 1911
697	*Ixodes woyliei* Ash, Elliot, Godfrey, Burmej, Abdad, Northover, Wayne, Morris, Clode, Lymbery and Thompson, 2017
698	*Ixodes zacateco* Apanaskevich and Bunn, 2025
699	*Ixodes zaglossi* Kohls, 1960
700	*Ixodes zairensis* Keirans, Clifford and Walker, 1982
701	*Ixodes zealandicus* Dumbleton, 1961
702	*Margaropus reidi* Hoogstraal, 1956
703	*Margaropus wileyi* Walker and Laurence, 1973
704	*Margaropus winthemi* Karsch, 1879
705	*Nosomma keralensis* Prakasan and Ramani, 2007
706	*Nosomma monstrosum* (Nuttall and Warburton, 1908)
707	*Rhipicentor bicornis* Nuttall and Warburton, 1908
708	*Rhipicentor nuttalli* Cooper and Robinson, 1908
709	*Rhipicephalus afranicus* Bakkes, 2018
710	*Rhipicephalus annulatus* (Say, 1821)
711	*Rhipicephalus appendiculatus* Neumann, 1901
712	*Rhipicephalus aquatilis* Walker, Keirans and Pegram, 1993
713	*Rhipicephalus armatus* Pocock, 1900
714	*Rhipicephalus arnoldi* Theiler and Zumpt, 1950
715	*Rhipicephalus aurantiacus* Neumann, 1907
716	*Rhipicephalus australis* Fuller, 1899
717	*Rhipicephalus bequaerti* Zumpt, 1950
718	*Rhipicephalus bergeoni* Morel and Balis, 1976
719	*Rhipicephalus boueti* Morel, 1957
720	*Rhipicephalus bursa* Canestrini and Fanzago, 1878
721	*Rhipicephalus camicasi* Morel, Mouchet and Rodhain, 1976
722	*Rhipicephalus capensis* Koch, 1844
723	*Rhipicephalus carnivoralis* Walker, 1966
724	*Rhipicephalus cliffordi* Morel, 1965
725	*Rhipicephalus complanatus* Neumann, 1911
726	*Rhipicephalus compositus* Neumann, 1897
727	*Rhipicephalus congolensis* Apanaskevich, Horak and Mulumba-Mfumu, 2013
728	*Rhipicephalus cuspidatus* Neumann, 1906
729	*Rhipicephalus decoloratus* Koch, 1844
730	*Rhipicephalus deltoideus* Neumann, 1910
731	*Rhipicephalus distinctus* Bedford, 1932
732	*Rhipicephalus duttoni* Neumann, 1907
733	*Rhipicephalus dux* Dönitz, 1910
734	*Rhipicephalus evertsi* Neumann, 1897
735	*Rhipicephalus exophthalmos* Keirans and Walker, 1993
736	*Rhipicephalus follis* Dönitz, 1910
737	*Rhipicephalus fulvus* Neumann, 1913
738	*Rhipicephalus geigyi* (Aeschlimann and Morel, 1965)
739	*Rhipicephalus gertrudae* Feldman-Muhsam, 1960
740	*Rhipicephalus glabroscutatus* Du Toit, 1941
741	*Rhipicephalus guilhoni* Morel and Vassiliades, 1963
742	*Rhipicephalus haemaphysaloides* Supino, 1897
743	*Rhipicephalus humeralis* Tonelli Rondelli, 1926
744	*Rhipicephalus hurti* Wilson, 1954
745	*Rhipicephalus interventus* Walker, Pegram and Keirans, 1995
746	*Rhipicephalus jeanneli* Neumann, 1913
747	*Rhipicephalus kadangbandi* Jeeran and Gambhir, 2020
748	*Rhipicephalus kochi* Dönitz, 1905
749	*Rhipicephalus kohlsi* (Hoogstraal and Kaiser, 1960)
750	*Rhipicephalus leporis* Pomerantzev, 1946
751	*Rhipicephalus linnaei* (Audouin, 1826)
752	*Rhipicephalus longiceps* Warburton, 1912
753	*Rhipicephalus longicoxatus* Neumann, 1905
754	*Rhipicephalus longus* Neumann, 1907
755	*Rhipicephalus lounsburyi* Walker, 1990
756	*Rhipicephalus lunulatus* Neumann, 1907
757	*Rhipicephalus maculatus* Neumann, 1901
758	*Rhipicephalus masseyi* Nuttall and Warburton, 1908
759	*Rhipicephalus microplus* (Canestrini, 1888)
760	*Rhipicephalus moucheti* Morel, 1965
761	*Rhipicephalus muehlensi* Zumpt, 1943
762	*Rhipicephalus muhsamae* Morel and Vassiliades, 1965
763	*Rhipicephalus neumanni* Walker, 1990
764	*Rhipicephalus nitens* Neumann, 1904
765	*Rhipicephalus oculatus* Neumann, 1901
766	*Rhipicephalus oreotragi* Walker and Horak, 2000
767	*Rhipicephalus pilans* Schulze, 1935
768	*Rhipicephalus planus* Neumann, 1907
769	*Rhipicephalus praetextatus* Gerstäcker, 1873
770	*Rhipicephalus pravus* Dönitz, 1910
771	*Rhipicephalus pseudolongus* Santos Dias, 1953
772	*Rhipicephalus pulchellus* (Gerstäcker, 1873)
773	*Rhipicephalus pumilio* Schulze, 1935
774	*Rhipicephalus punctatus* Warburton, 1912
775	*Rhipicephalus pusillus* Gill Collado, 1936
776	*Rhipicephalus ramachandrai* Dhanda, 1966
777	*Rhipicephalus rossicus* Yakimov and Kohl-Yakimova, 1911
778	*Rhipicephalus rutilus* Koch, 1844
779	*Rhipicephalus sanguineus* (Latreille, 1806)
780	*Rhipicephalus scalpturatus* Santos Dias, 1959
781	*Rhipicephalus schulzei* Olenev, 1929
782	*Rhipicephalus sculptus* Warburton, 1912
783	*Rhipicephalus secundus* Feldman-Muhsam, 1952
784	*Rhipicephalus senegalensis* Koch, 1844
785	*Rhipicephalus serranoi* Santos Dias, 1950
786	*Rhipicephalus simpsoni* Nuttall, 1910
787	*Rhipicephalus simus* Koch, 1844
788	*Rhipicephalus sulcatus* Neumann, 1908
789	*Rhipicephalus supertritus* Neumann, 1907
790	*Rhipicephalus tetracornus* Kitaoka and Suzuki, 1983
791	*Rhipicephalus theileri* Bedford and Hewitt, 1925
792	*Rhipicephalus tricuspis* Dönitz, 1906
793	*Rhipicephalus turanicus* Pomerantzev, 1940
794	*Rhipicephalus walkerae* Horak, Apanaskevich and Kariuki, 2013
795	*Rhipicephalus warburtoni* Walker and Horak, 2000
796	*Rhipicephalus zambeziensis* Walker, Norval and Corwin, 1981
797	*Rhipicephalus ziemanni* Neumann, 1904
798	*Rhipicephalus zumpti* Santos Dias, 1950
799	*Robertsicus elaphensis* (Price, 1959)
800	*Sharifiella theilerae* (Hoogstraal, 1953)

Genera and species are listed alphabetically, and fossil species are marked with an asterisk (*)

The fossil species originally described as *Haemaphysalis cretacea* Chitimia-Dobler, Pfeffer and Dunlop, 2018 has been debated regarding its placement within the genus *Haemaphysalis* [18–20]. The species has recently been redescribed as *Alloceraea cretacea* (Chitimia-Dobler, Pfeffer and Dunlop, 2018), and a new fossil nymphal specimen has been added to that species [63]. This species is, therefore, retained in the current list as a valid species in the genus *Alloceraea*.

## The families Khimairidae and Nuttalliellidae: ancient tick lineages

The family Khimairidae, the most recently described tick family, includes only one genus and one fossil species, namely, *Khimaira fossus* Chitimia-Dobler, Mans, and Dunlop, 2022 [24]. In contrast, the family Nuttalliellidae, which was formerly monospecific, now comprises three genera and 11 species [25]. Table 4 summarizes the currently recognized species in the family Nuttalliellidae. Notably, all species in these families are fossils, except for *Nuttalliella namaqua* Bedford 1931, which is regarded as a living fossil and the only known living member of the most primitive tick lineages [3, 16, 64, 65].

## How many ticks are still out there to be discovered?

**Fig. 1:**
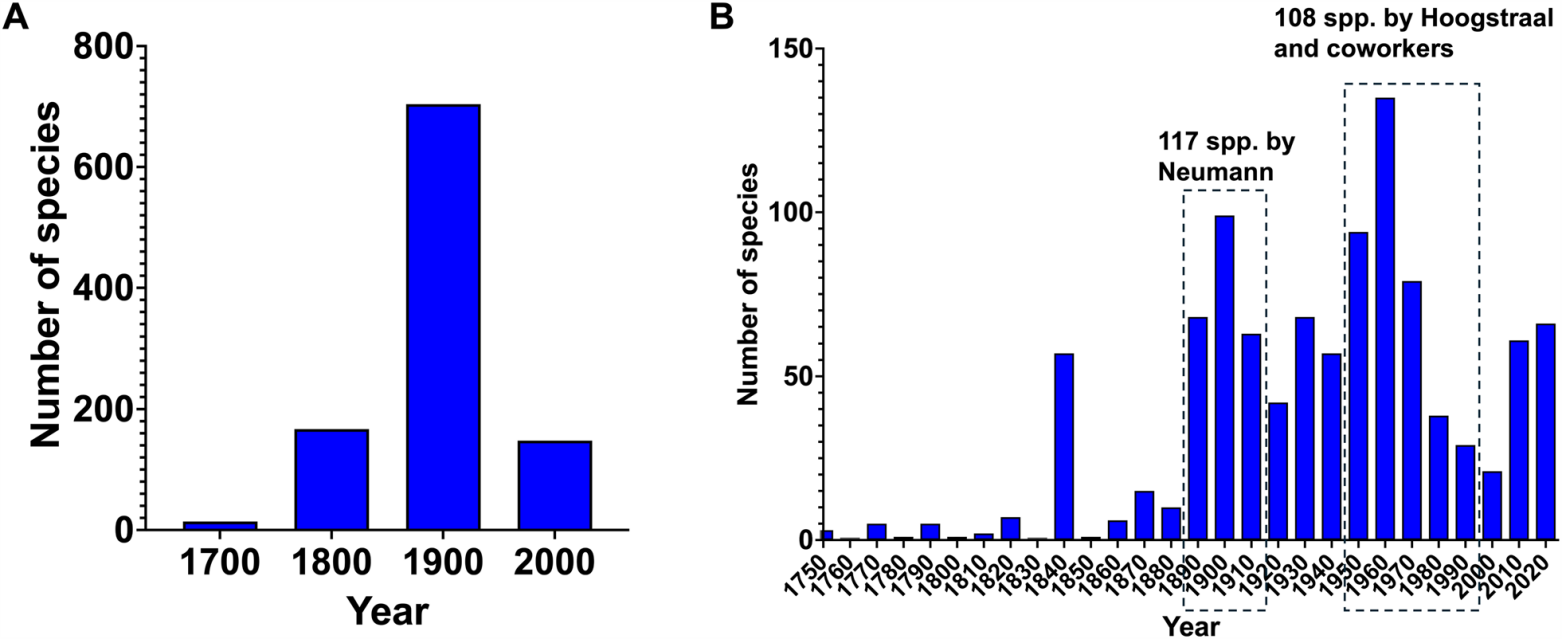
Number of species described in the 1700s, 1800s, 1900s, and 2000s (**A**). Number of species described per decade, highlighting the counts for the two most prominent tick taxonomists of our time: Louis Georges Neumann and Harry Hoogstraal (**B**). Graphs were prepared with GraphPad Prism for macOS (Version 11.0.0)

Among the valid tick species listed here (*n* = 1033), most (68.2%) were described in the 1900s (Fig. 1A). Of these, 81.8% were described by 10 leading tick taxonomists: Louis G. Neumann (117), Harry Hoogstraal (108), Glen M. Kohls (89), Carleton M. Clifford (60), Cecil Warburton (41), Don Ramsay Arthur (36), James E. Keirans (36), Paul Schulze (33), George H. F. Nuttall (30), and Robert A. Cooley (26). Two of them described more than 100 valid tick species: Louis G. Neumann and Harry Hoogstraal (Fig. 1B; Table 4).

**Table 4:** List of valid species in the family Nuttalliellidae

No	Species
1	*Deinocroton bicornis* Chitimia-Dobler, Dunlop and Mans, 2024*
2	*Deinocroton copia* Chitimia-Dobler, Mans and Dunlop, 2022*
3	*Deinocroton draculi* Peñalver, Arillo, Anderson and Pérez-de la Fuente, 2017*
4	*Deinocroton lacrimus* Chitimia-Dobler, Dunlop and Mans, 2024*
5	*Legionaris robustus* Chitimia-Dobler, Dunlop and Mans, 2024*
6	*Nuttalliella gratae* Chitimia-Dobler, Dunlop and Mans, 2024*
7	*Nuttalliella namaqua* Bedford, 1932
8	*Nuttalliella odyssea* Chitimia-Dobler, Dunlop and Mans, 2024*
9	*Nuttalliella placaventrala* Chitimia-Dobler, Dunlop and Mans, 2024*
10	*Nuttalliella tropicasylvae* Chitimia-Dobler, Dunlop and Mans, 2024*
11	*Nuttalliella tuberculata* Chitimia-Dobler, Dunlop and Mans, 2024*

Fossil species are indicated with an asterisk (*)

**Fig. 2:**
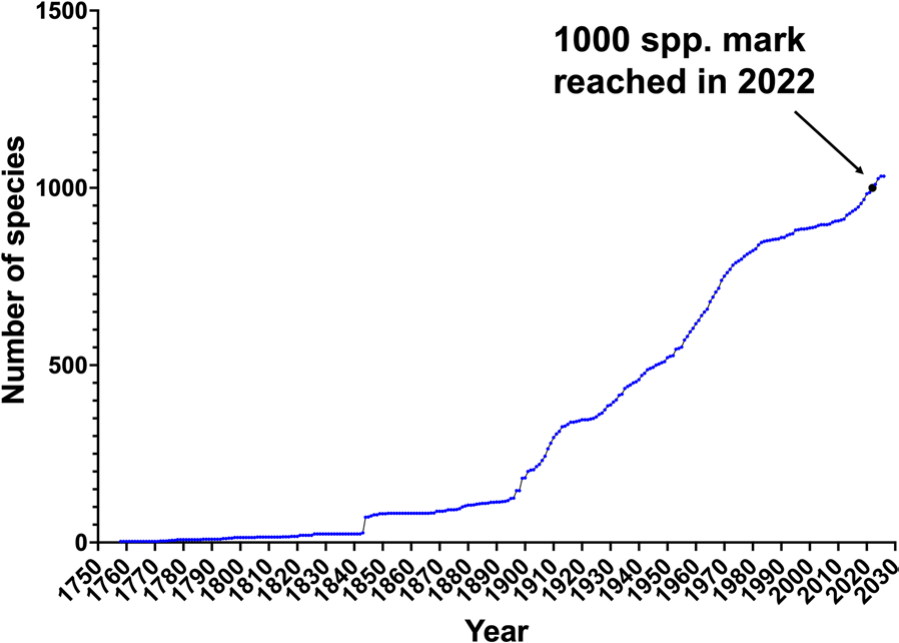
Cumulative number of species described from 1758 to 2026. This graph was prepared using GraphPad Prism for macOS (Version 11.0.0)

Predicting the number of remaining tick species is difficult, but from 2000 to 2025, 148 valid species were described, which is significant. To put this into perspective, during the golden age of tick taxonomy, 173 species were described from 1900 to 1925. This is clear from the observed upward trend (Fig. 2), which suggests that the current rate of new species descriptions is comparable to rates from bygone eras.

**Fig. 3:**
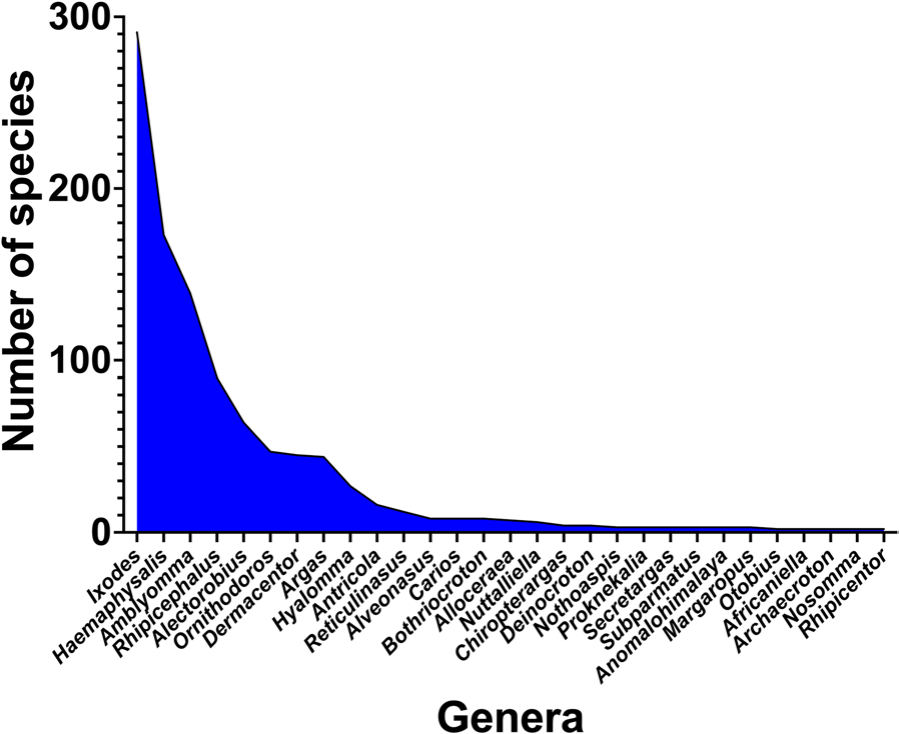
Number of species by tick genus. Monospecific genera (*Apanaskevichiella*, *Australpavlovskyella*, *Compluriscutula*, *Cornupalpatum*, *Cosmiomma*, *Cryptocroton*, *Khimaira*, *Legionaris*, *Navis*, *Ogadenus*, *Robertsicus*, and *Sharifiella*) are not depicted. This graph was prepared using GraphPad Prism for macOS (Version 11.0.0)

The genus *Ixodes* is the most speciose (Fig. 3), with 291 species, 47 of which were described between 2000 and 2025. During the same period, 16 and 13 species of *Haemaphysalis* and *Amblyomma* were described, respectively. For the family Argasidae, 19 species of *Alectorobius* and 13 of *Ornithodoros* were described in the same period.

Although fewer species were described in the early 2000s than in the early 1900s, advances in molecular biology are accelerating the discovery of new species, and modern microscopy also aids species identification. It is reasonable to assume that many more species remain to be discovered, especially in countries and regions, where few studies have been conducted.

## Conclusions

Tick systematics has undergone several changes in recent years, primarily driven by molecular phylogenetic studies, particularly those based on mitogenomes. The most significant changes occurred within the family Argasidae, resulting in the establishment of several new genera. This review provides an updated list of valid tick genera and species worldwide. We hope this list will benefit tick researchers and inspire further studies of this important group of parasites.

## Data Availability

Data supporting the conclusions of this review are included in the manuscript text and tables.
